# Enhanced production of a recombinant xylanase (XT6): optimization of production and purification, and scaled-up batch fermentation in a stirred tank bioreactor

**DOI:** 10.1038/s41598-023-48202-5

**Published:** 2023-11-28

**Authors:** Priyashini Dhaver, Tariro Sithole, Brett Pletschke, Bruce Sithole, Roshini Govinden

**Affiliations:** 1 Discipline of Microbiology, School of Life Sciences, University of KwaZulu-Natal, Westville Campus, Durban, 4000 South Africa; 2 Enzyme Science Programme (ESP), Department of Biochemistry and Microbiology, Rhodes University, Makhanda (Grahamstown), 6140, Eastern Cape South Africa; 3 Biorefinery Industry Development Facility, Council for Scientific and Industrial Research, Durban, 4000 South Africa; 4 Discipline of Chemical Engineering, University of KwaZulu-Natal, Durban, 4000 South Africa

**Keywords:** Biotechnology, Microbiology

## Abstract

The endoxylanase XT6 produced by *Geobacillus stearothermophilus* is a desirable candidate for industrial applications. In this study, the gene encoding XT6 was cloned using the pET-28a expression vector and expressed in *Escherichia coli* BL21 (DE3) cells. Recombinant XT6 production was improved by optimizing cell lysis (sonication, chemical, and enzymatic lysis) and expression conditions. Sonication in a 0.05 M sodium phosphate (pH 6.0) buffer resulted in the highest xylanase activity (16.48 U/ml). Screening and optimization of induction conditions using the Plackett–Burman Design and Box-Behnken Design (BBD) approaches revealed that cell density pre-induction (OD_600 nm_), post-induction incubation time, and IPTG concentration significantly (*p* < 0.05) influenced the expression levels of XT6 (16.48 U/ml to 40.06 U/ml) representing a 3.60-fold increase. BBD resulted in a further 8.74-fold increase in activity to 144.02 U/ml. Batch fermentation in a 5-l stirred tank bioreactor at 1 vvm aeration boosted recombinant xylanase production levels to 165 U/ml suggesting that heterologous expression of the XT6 enzyme is suitable for scaled-up production. The pure enzyme with a molecular weight of 43 kDa and a 15.69-fold increase in purity was obtained using affinity chromatography and a cobalt column. Future studies will include application of the purified recombinant xylanase to animal feed.

## Introduction

Problem statement: Interest in xylan-degrading enzymes has escalated over the last few years due to their applications in pulp processing and the biodegradation of lignocellulosic materials^1^. Hemicellulose, which makes up 30–40% of lignocellulosic biomass, is mainly constituted of xylan^1^. Xylan is a polysaccharide made up of β-1,4-xylose units or β-1,4-mannose units with arabinose, methylglucuronic acid, and acetate substitutions^2,3^. The complex chemical composition of xylan requires the concerted action of several enzymes, collectively known as hemicellulases, including endo-β-D-xylanases, β-xylosidases, α-L-arabinofuranosidases, α-D-glucuronidases, acetyl xylan esterases, ferulic and *p*-coumaric acid esterases. These enzymes work together to produce xylooligosaccharides (XOS) and xylose, which are the end products of their synergistic action on the linear and side chains^4^. Endo-1,4-β-xylanase is an important enzyme that operates on the xylan backbone of hemicellulose with high specificity, minimum substrate loss, and few side products^2^ compared to the commonly used chemical hydrolysis techniques. Xylanase acts synergistically in conjunction with other accessory enzymes to degrade xylan to component sugars.

Current state of the art: In nature, filamentous fungi such as *Aspergillus* spp.^5^ and *Trichoderma* spp.^6^ produce xylanases rapidly. Some bacteria, such as *Bacillus stearothermophilus, Bacillus subtilis*^7^, and *Paenibacillus* spp., produce extracellular thermostable xylanase enzymes^8^. These thermostable xylanases are more suitable for industrial bioprocesses than those produced by mesophilic bacteria^3^. The genus *Geobacillus* has been widely examined among numerous thermophilic bacteria studied for xylanase production due to its ability to produce highly thermostable enzymes and ability to use a variety of carbon sources^9^. In thermophilic niches, the genus evolved several species, whose genomes encode highly thermostable enzymes that can be applied in several industrial bioprocesses such as in lignocellulosic biomass hydrolysis^6^, pulp and paper production^10^, bioethanol production^11^, etc. In recent years, xylanases with specific properties have been identified from bacterial and fungal sources, and numerous strategies have been developed to engineer xylanases for specific industrial applications^12^. Due to product inhibition and catabolite repression, microbial xylanases are produced in low titres.

Recent proposed strategies: Researchers have introduced recombinant DNA technology to solve this challenge which has led to the development of xylanolytic enzymes that are more appropriate for industrial applications. To attain overproduction of the enzyme to suit commercial purposes, the xylanase-encoding genes have been cloned into homologous and heterologous hosts^13^. *Escherichia coli* is the first-line system for the heterologous production of particularly non-glycosylated proteins^14^. Cloning of genes in *E. coli* is considered advantageous for high enzyme production levels as it can be easily grown and rapidly on inexpensive substrates to high cell density under favorable growth conditions to achieve high-level expression of the desired recombinant protein^15^. Moreover, *E. coli* can be easily manipulated and has several available cloning and expression vectors, and is amenable to various cultivation techniques^14^. In view of these advantages, *E. coli* can be a suitable host for the large-scale manufacturing of heterologous proteins^14^ in bioreactors that provide a well-controlled culture environment^16^. One of the most significant disadvantages of using *E. coli* to produce desired products is that these bacteria do not ordinarily release proteins into the environment^17^. Proteins produced remain confined within the constraints of the cellular framework requiring disruption of the cell walls for the release of proteins into the surrounding environment. Techniques such as sonication, chemical lysis, enzymatic lysis, bead milling, and high-pressure homogenization are reported to be effective for the recovery of proteins from *E. coli* cells^18^.

The reaction of cells to their surroundings can also influence the host’s expression level^19^. As a result, fermentation factors such as temperature, cell density, induction period, and inducer concentrations must be optimized for the host cells, to maintain favorable conditions to ensure high enzyme activity and protein production efficiency^14^. The traditional method for optimizing the fermentation process is the one-factor-at-a-time (OFAT) approach, where one parameter is varied, while the others are kept constant. However, this approach is laborious due to many factors and the inability to identify the interactions between variables, which can cause misinterpretation of the results^20^. Such challenges may be overcome by using statistical approaches. Plackett Burman Design (PBD)  and Rresponse Ssurface Methodology (RSM) approaches were used in this study to optimize the expression of the *G. stearothermophilus* XT6 in *E. coli* to produce high yields of the functional recombinant XT6 protein. Significant steps in the production of a recombinant protein after cloning include fermentation to produce high biomass yields, cell lysis to release intracellular proteins, and the recovery of the protein of interest using a targeted separation technique such as Immobilized Affinity Chromatography (IMAC). All steps are paramount for the success and economic viability of the process.

Aims and objectives: This study aimed to optimize cell lysis (sonication, chemical lysis, and enzymatic lysis) which is critical to achieving the highest possible yield of soluble protein, the expression of XT6 by *E. coli* in shake flask studies, and the scaled-up of production in large-scale bioreactors. The expressed recombinant XT6 protein was then purified and used in further application studies to improve the digestibility of animal feed and assess the effect of the recombinant XT6 xylanase on the hydrolysis of feed substrates.

## Materials and methods

### Obtaining *E. coli* with pET28(+)/XT6 and expression of recombinant XT6 xylanase

*E. coli* BL21(DE3) cells were transformed with the pET28(+)-XT6 plasmid DNA^22^. The recombinant cells were grown on 2 × YT plates containing 50 µg/ml kanamycin (SIGMA, China) and were incubated (Heraeus B6120 Incubator, Gemini BV) for 24 h at 37 °C^22^.

Single colonies of the recombinant *E. coli* cells harboring XT6 were cultured in 5 ml of 2 × YT broth (50 µg/ml kanamycin) and incubated at 37 °C for 24 h. The culture was transferred into fresh 2 × YT broth (50 µg/ml kanamycin) and grown at 37 °C until the mid-log phase at OD_600 nm_ was between 0.4 and 0.7. Protein expression was induced by the addition of 1 mM isopropyl-β-Dthiogalactopyranoside (1 M IPTG) (Glentham Life Science, Corsham). Samples were taken every hour for up to 4 h, and the OD_600 nm_ readings of the bacterial cells were recorded each hour. Collected samples were centrifuged (Eppendorf centrifuge 5418, Germany) at 16 060×*g* for a minute. The supernatant was discarded, and the pellet resuspended in 2 × SDS sample buffer (0.004% (v/v) bromophenol blue, 10% (v/v) 2-mercaptoethanol, 20% (v/v) glycerol, 4% (v/v) SDS and 0.125 mM Tris-HCl, Sigma, South Africa). The volume of sample buffer used to resuspend the pellet was obtained using the formula: [resuspension volume (ml) = OD_600 nm_/6]. Samples were boiled for 5 min and then incubated on ice before SDS-PAGE analysis^22^. All chemicals and reagnts were obtained from Sigma, Merck.

### Optimization of cell lysis

Lysis is a critical stage in the purification of intracellular bioproducts. There are several factors and challenges to consider when selecting the correct lysis protocol as there is no standard procedure applicable for the recovery of all types of recombinant proteins^18^. It is thus, advisable to thoroughly study and test various lysis protocols to ensure the highest possible recovery of the desired product. The different lysis protocols that were tested to maximize recovery of the intracellular recombinant xylanase from the *E. coli* expression host are described below.

#### Enzymatic lysis (lysozymes)

After induction, the cells were harvested by centrifugation (Eppendorf Centrifuge 5418, Germany) (10,000×*g* at 4 °C for 10 min), and the pellet was resuspended in phosphate-buffered saline (PBS) buffer (20 ml/g of cells). Lysozyme (1 mg/ml) (Sigma, Switzerland) was added to the cell suspension and incubated with shaking at room temperature for 2 h. Samples were stored at − 80 °C overnight and thereafter centrifuged as described above, and the protein was obtained in the supernatant (soluble fraction)^18^.

#### Enzymatic and chemical lysis

The procedure described above in 2.3.1.1 was followed. However, TritonX-100 (1% v/v) (Merck chemicals, England) was used in conjunction with lysozyme, as TritonX-100 is reported to assist in the disruption of the cellular membrane leading to enhanced cell lysis^23^.

#### Sonication

The harvested cells were centrifuged, and the pellets resuspended in 0.05 M sodium phosphate (pH 6.0), 0.05 M Tris–HCl (pH 8.0) buffer, and 0.05 M Tris-HCl (pH 8.0) with 8 M urea. The cell suspensions were lysed using a probe sonicator (OMNI Sonic Ruptor 400, 220 V 6A, 18-200, United Kingdom) at 50 kHz for 30 s. The samples were kept on ice to prevent heating and denaturation during sonication. The lysate was centrifuged for 10 min at 10,000×*g* at 4 °C.  The soluble fraction was expected to contain the target protein^18^.

### Quantification of the extent of lysis

There are several techniques which can be used to quantify the extent of cell lysis. These can be categorized as direct and indirect analyses of cellular lysis.

#### Direct cellular analysis: optical density at 600 nm

The extent of the lysis of *E. coli* cells can be determined by measuring and comparing the OD_600 nm_ before and after cell lysis treatments^24^.

#### Indirect analysis (quantification of cellular products)

The indirect techniques of cellular lysis quantification are based on separating several cellular products resulting from cell lysis.

##### Quantification of total protein

The total protein content was determined using the Bradford technique using bovine serum albumin (BSA) (Sigma, USA) as the standard at values ranging from 0 to 50 mg/ml. In a reaction vessel, aliquots of 1 ml Bradford reagent (Sigma, USA) were well mixed with 33.33 μl of protein and allowed to stand for 5 min at room temperature. A spectrophotometer was used to detect the absorbance at 595 nm (Shimadzu UV-1800, Japan). The blank was comprised of 33.33 μl of distilled water mixed with Bradford reagent^26^. The degree of cell lysis was evaluated by measuring the concentration of proteins in the supernatant of lysate samples before and after lysis.

##### Xylanase protein quantification

The concentration of the xylanase protein was determined before and after cell lysis using a spectrophotometer (Nanodrop 2000c spectrophotometer, Thermo Scientific). The molar extinction coefficient used was 80 790 M^−1^ cm^−1^ at 280 nm, with a theoretical molecular weight of 46 763 Da for the enzyme^25^.

##### Quantification of xylanase activity

Xylanase activity was quantified using the 3,5-dinitro salicylic (DNS) (Sigma, India) acid assay for reducing sugars^27^. The reaction included 600 µl of 1% (w/v) of beechwood xylan (1 g in 100 ml of citrate buffer, pH 5) (Sigma, India), to which 66.67 µl of the enzyme was added and incubated in a water bath at 55 °C for 15 min. The reaction was terminated by adding 1 ml DNS acid reagent to the reaction mixture and then heated for 5 min at 100 °C in a water bath. The absorbance was read at 540 nm using a spectrophotometer (Shimadzu UV-1800, Japan) to determine the concentration of sugar released by the enzyme. One unit (U) of xylanase was defined as the amount of enzyme that released 1 µmol xylose as reducing sugar equivalents per min under the specified assay conditions. All enzyme assays were performed in triplicate.

##### SDS-PAGE

The molecular weight of the recombinant XT6 xylanase was confirmed by 12% SDS-PAGE^28^. The gel was run at a constant voltage (50 V) (BioRad, Power Pac™ HV, USA) until the dye front reached the bottom of the gel. Following electrophoresis, the gel was stained in Coomassie Brilliant Blue (Merck, Germany) for 15 min and destained overnight in a destaining solution (10% acetic acid (Merck, Germany), 20% methanol (Merck, Germany), and 70% dH_2_0) to visualize the proteins and determine the molecular weight of the proteins using standard molecular weight markers. To determine the degree of lysis samples representing the total lysate proteins, both the insoluble phase (pellet), and lysate supernatant (soluble phase) were run on the gel The greater the number of proteins (represented by the number of bands as well as the intensity of bands) released to the soluble phase, the greater the cellular lysis efficiency.

### Experimental design and optimization of cultivation parameters

#### OFAT optimization of the production of the recombinant XT6 xylanase

The factors tested included cell density pre-induction (OD_600 nm_)^31^, induction temperature^30^, time^13^, IPTG concentration^13^, as well as yeast extract, and tryptone concentration^29^.

#### Statistical optimization, experimental design, and data analysis

##### Plackett–Burman design (PBD)

Six variables were selected for this study as shown in Table 1: Incubation temperature (X1), cell density pre-induction (OD_600 nm_) (X2), post-induction time (X3), yeast extract concentration (X4), tryptone concentration (X5), and IPTG concentration (X6). The total number of experimental runs carried out for the six variables was twelve^32^. Each variable was represented by a high level denoted by ‘+’ and a low level denoted by ‘−’. The high level of each variable was sufficiently far from the low level so that any significant effect would be observed. The experimental runs were performed in duplicate, and an average of the results was reported. Table 1 represents the PBD based on the first-order polynomial model Eq. (1).

1 $${\text{Y}} =\upbeta _{0} \sum\upbeta _{{\text{i}}} {\rm X}$$

where Y is the response (peak area and retention factor), β_0_ is the model intercept, βi is the linear coefficient, and Χ_i_ is the level of the independent variable. The PBD was analyzed using R studio software^33^ to estimate the significant factors. Analysis of variance (ANOVA) was performed to determine the *p* values and the *R* coefficients to check the significance and fit of the regression model. Screened parameters were represented on a Pareto chart of standardized effects. The effect of each variable was analyzed, and the variables with the highest influence on the production of xylanase were selected for the second-level optimization by BBD of Response Surface Methodology (RSM).

**Table 1:** Experimental variables and levels used in the Plackett Burman Design for optimal recombinant XT6 xylanase production.

Symbol code		Units	Experimental values	
			Low level (− 1)	High level (+ 1)
Induction temperature	X1	°C	25	37
Cell density pre-induction (OD_600 nm_)	X2	–	0.4	0.6
Post induction time	X3	Hours (h)	3	5
Yeast extract	X4	%	0.5	1.5
Tryptone	X5	%	1	2
IPTG	X6	mM	0.5	2.5

##### Optimization of significant variables using Response Surface Methodology (RSM)

The BBD was used to elucidate the primary interaction and quadratic effects of the three significant variables arising from the PBD, with replicated centre points. The experimental design and statistical analysis were performed using R Studio^33^. Table 2 represents a three-level, three-factor BBD was used to evaluate the combined effect of the three independent variables, cell density pre induction (OD_600 nm_) (X2), post-induction time (X3), and IPTG concentration (X6). The design consisted of 16 combinations, including three replicates of the centre point. After the experimental runs, the average xylanase activities were taken as the response (Y). A multiple regression analysis of the data was carried out to obtain an empirical model relating the response to the independent variables.

**Table 2:** Experimental codes and levels of independent variables in the RSM for optimal recombinant XT6 xylanase production.

Variables	Symbol code	Experimental values		
		Lower (− 1)	Zero (0)	Higher (+ 1)
Cell density pre-induction (OD_600 nm_)	X2	0.4	0.5	0.6
Post-induction time (h)	X3	3	4	5
[IPTG] (mM)	X6	0.5	1.5	2.5

The second-order polynomial equation is shown below in Eq. (2):

2 $${\text{Y}} =\upbeta _{0} + \sum\upbeta _{{\text{i}}} {\rm X}_{{1}} + \, \sum\upbeta _{{{\text{ii}}}} {\rm X}_{{2}} + \sum\upbeta _{{{\text{ij}}}} {\rm X}_{{1}} {\text{X}}_{{2}}$$

where Y represents the response variable (peak area), β_0_ is the interception coefficient, β_i_ is the coefficient for the linear effects, β_ii_ is the coefficient for the quadratic effect, β_ij_ are interaction coefficient, and Χ_1_ Χ_2_ is the coded independent variables that influence the response variable Y. The response in each run was the average. In this experimental design, data were analyzed by one-way ANOVA with Tukey’s multiple comparison tests (*p* < 0.05) using R studio^33^, and ggplot2 was used for the generation of 3D response surface and contour plots.

### Scaled-up enzyme production in a stirred tank bioreactor

This study was carried out in a Sartorius BioStat®B-DCU fermenter with a working volume of 3-l with 2 × YT medium supplemented with kanamycin (50 µg/ml) in a UniVessel^®^ Glass 5 L (260 mm diameter and 690 mm height). The fermenter was sterilized at 121 °C for 15 min. Optimal conditions from the lab-scale production were used to set up fermentation in the bioreactor. A BioStat^®^B twin control tower with MFCS was used to monitor all the relevant parameters and data. The pH and Dissolved Oxygen (DO) probes were first calibrated according to the standard procedure given by the manufacturer. Fermentation was carried out at 30 °C, 200 rpm with one impeller (Rushton blade disc impeller). Three different aeration rates: 0.5, 1, and 2 volume of air per volume of liquid per min (vvm) were tested. Growth was monitored every 30 min and after 2 h, cells reached the expected OD_600 nm_ (0.5), and IPTG was added to the fermentation. After 4 h of fermentation, the content of the bioreactor was harvested, and down streaming was carried out with the separation of the pellet and supernatant using the centrifuge (Beckman Coulter™, Avanti^®^ J-26XPI, USA) at 4 °C, 16 873×*g* for 10 min.

#### Specific growth rate

The specific growth rate is the most important parameter to be determined during fermentation, as it represents the dynamic behavior of microorganisms. The specific growth rate period is defined as the rate of increase of biomass of a cell population per unit of biomass concentration. This can be determined by obtaining the gradient of the growth curve shown in Fig. 8B^34^, Eq. (3).

3 $${\text{Specific}} \,{\text{growth}}\,{\text{ rate}} \left(\upmu \right) = \frac{{ {\text{ODx}} {-} {\text{ODy}}}}{{{\text{Tx}} - {\text{Ty}}}}$$

#### Productivity

Productivity is defined as the final product concentration divided by the time from inoculation to batch delivery. This is determined by the final biomass concentration subtracted from the inoculum concentration divided by the cultivation time (h), Eq. (4).

4 $${\text{P}}_{{\text{o}}} = \left( {{\text{X}}_{{\text{F}}} - {\text{ X}}_{{\text{O}}} } \right)/{\text{t}}_{{\text{c}}}$$

where X_F_ is the final biomass concentration (g/l), X_o_ is the inoculum (g/l), and t_c_ is the cultivation time (h).

#### Biomass yield coefficient

The biomass yield coefficient could be defined as the mass of microorganisms produced per mass of a substrate utilized, known as the growth yield coefficient^35^, Eq. (5).

5 $${\text{Yield coefficient}} = \;\frac{\text{Biomass produced}}{\text{Substrate utilized}}$$

### Purification of recombinant XT6 xylanase

Purification was conducted using affinity chromatography. A column was packed with the appropriate amount of HisPur cobalt resin, and gravity flow allowed the storage buffer to drain from the resin. Two resin bed volumes of the equilibration/wash buffer (50 mM NaH_2_PO_4_, 300 mM NaCl, 0.03% (w/v) sodium azide, 10 mM imidazole, and 50 mM Na_2_HPO_4_, pH 8.0) were added. The buffer was allowed to drain from the resin at a flow rate of 0.5–1 ml/min. Two resin bed volumes of the prepared protein extract (supernatant) were loaded directly into the column containing the HisPur cobalt resin (SIGMA, USA). The flow-through was collected and reapplied to maximize the yield. The supernatant was decanted and kept as the flow-through fraction (FT-unbound protein sample). The resin was washed with two resin-bed volumes of equilibration/wash buffer to remove all non-specifically bound proteins on the resin. This was repeated until the absorbance of the flow-through fraction, at 280 nm, reached baseline. The flow-through was collected each time in a new collection tube and labeled as the “wash” fractions (W1–W3). Two-resin bed volumes of elution buffer (50 mM NaH_2_PO_4_, 300 mM NaCl, 0.03% (w/v) sodium azide, 250 mM imidazole, and 50 mM Na_2_HPO_4_, pH 8.0, SIGMA, USA) were added to the resin and repeated three times (E1–E3), to elute His-tagged proteins and any remaining protein. A final wash step (W4) was conducted to remove residual imidazole using the wash buffer from the column. The three elution fractions were pooled together and concentrated using 30 kDa Amicon filters by centrifugation (4000×*g* at 4 °C for 20 min) (Eppendorf centrifuge 5418, Germany). The concentrate was then constituted in a final glycerol concentration of 20% (v/v) for XT6 stabilization during storage at -20°C. To store the cobalt column appropriately for regeneration, it was washed with ten resin-bed volumes of 20 mM 2-(N-morpholino)ethanesulfonic acid (MES) buffer (SIGMA, South Africa), 0.05 M NaCl, pH 5.0, followed by ten resin-bed volumes of ultrapure water and stored in 30% ethanol at 4 °C.

## Results and discussion

### Expression of the recombinant XT6 xylanase

**Figure 1:**
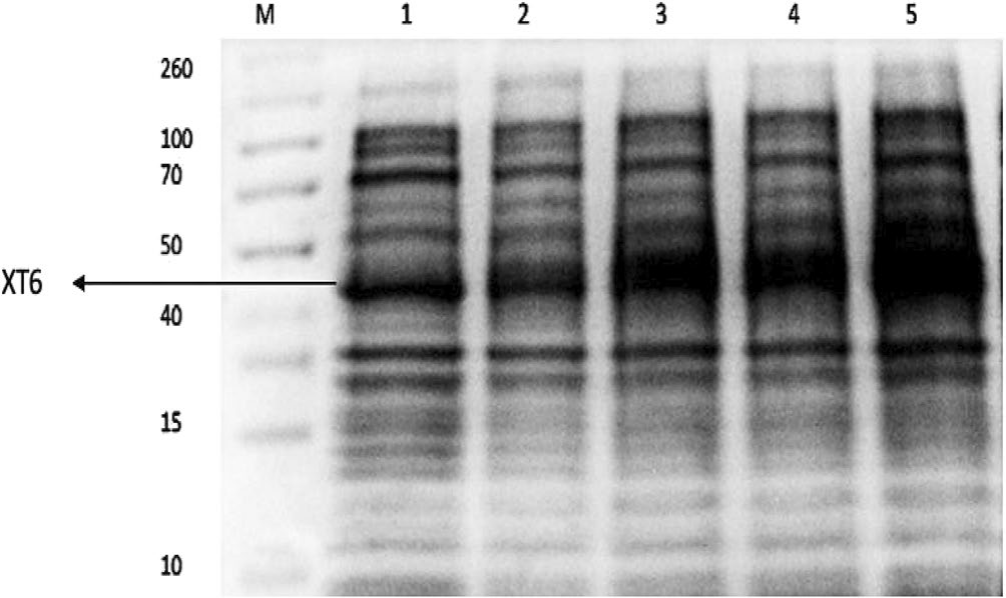
12% SDS-PAGE gel showing expression of the recombinant *G. stearothermophilus* xylanase (XT6) in *E. coli* BL21 (DE3) cells. Lane M: Molecular weight marker (Thermo Scientific, USA), Lane 1: uninduced sample, and Lanes 2–5: Induction samples at 1–4 h, respectively. (Original image shown in supplementary Fig. 1).

Induction and expression of the recombinant XT6 xylanase were performed by growing the cells until the OD_600 nm_ was between 0.4 and 0.6, then adding 1 mM IPTG, followed by hourly sampling (1 to 4 h). The insoluble fractions were analyzed using 12% SDS-PAGE and illustrated in Fig. 1, with the uninduced cell lysate serving as a control. More highly contrasted bands of 43 kDa were observed in the cell lysate of samples after induction, while less contrasted bands were observed for the control (uninduced), indicating successful expression of the cloned XT6 gene. Gomez Garcia et al. reported a similar molecular weight (45 kDa) for a xylanase from *Geobacillus* sp. DUSELR13^6^.

### Optimization of cell lysis

**Figure 2:**
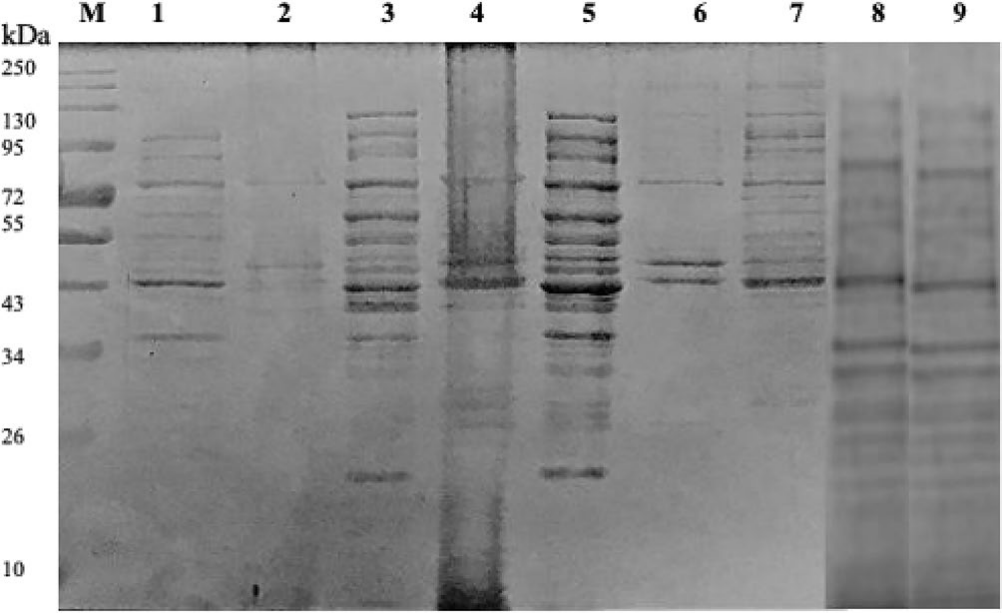
12% SDS-PAGE gel showing the recombinant XT6 expressed in *E. coli* BL21 (DE3) cells after various lysis techniques. Lane M: Molecular weight marker (Thermo Scientific, USA), Lane 1: uninduced sample, Lanes 2 and 3: insoluble and soluble fractions after lysis with lysozyme + 1% TritonX-100, Lanes 4 and 5: insoluble and soluble fractions following sonication in 0.05 M sodium phosphate (pH 6.0) buffer, Lane 6: insoluble fraction sonicated in 0.05 M Tris–HCl and 8 M urea, Lane 7: soluble fraction sonicated in Tris–HCl buffer, and Lanes 8 and 9: insoluble and soluble fraction treated with lysozyme. (Original image shown in supplementary Fig. 2).

**Figure 3:**
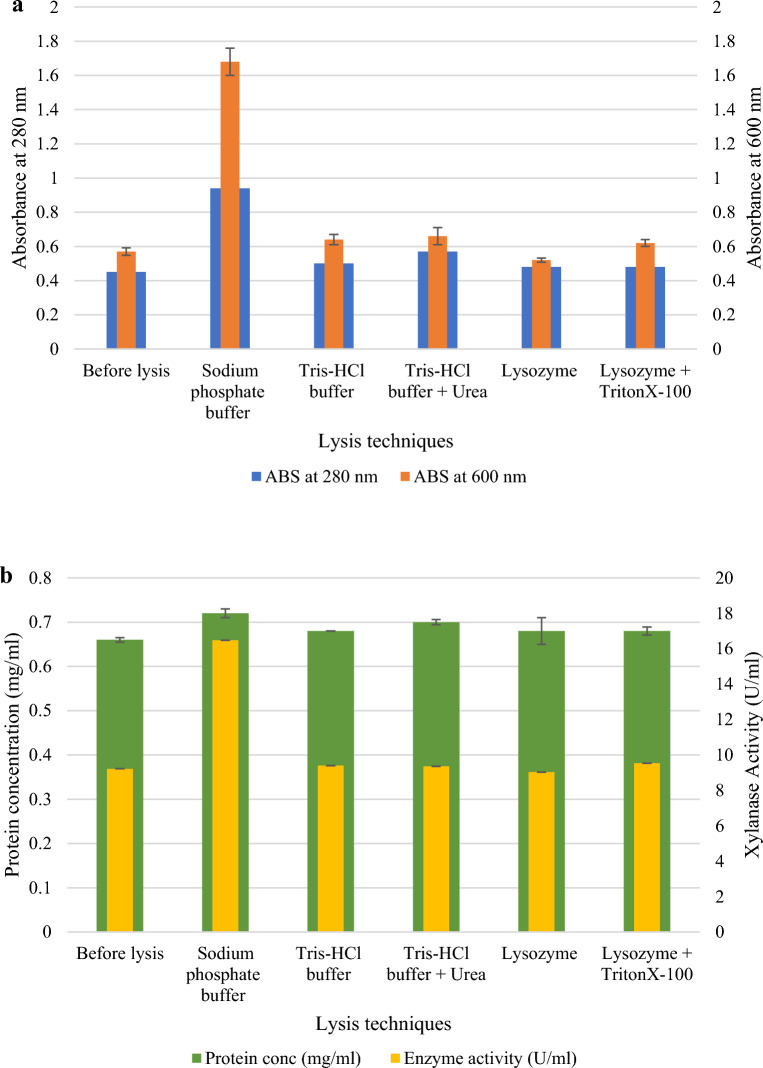
Analysis of the lysis methods to assess the efficiency of lysis of recombinant *E. coli* cells expressing the XT6 xylanase representing (**a**) OD_280 nm_ and OD_600 nm_, and (**b**) protein concentration, and xylanase activity after different lysis techniques.

A series of experiments were performed to determine the most efficient method for lysing the recombinant *E. coli* cells. The SDS-PAGE gel in Fig. 2 showed that sonication with a 0.05 M sodium phosphate (pH 6.0) buffer resulted in the highest protein concentrations compared to the other lysis procedures. Figure 3 shows the various direct and indirect analysis methods to monitor cell lysis. Sonication with 0.05 M sodium phosphate buffer (pH 6.0) was effective as higher enzyme activity was obtained after cell lysis (16.48 U/ml) compared to the other techniques (9.40, 9.36, 9.03, and 9.53 U/ml) (*p* < 0.05). There was a significant difference (*p* < 0.05) between the “Before” and “After” cell lysis fractions for each analysis and method of lysis.

Sonication has been shown to be an effective method for lysing bacterial cell walls^21^. Sonication, using a probe to generate sound energy, usually within a range of 20–50 kHz, can disrupt the structure of cells through the formation of violent implosions of small bubbles and cavitation within the sample. The energy of the sonic waves can disrupt the intramolecular forces that provide the integrity for the cellular wall^21^. Tris-HCl has an effective pH range of 7.0 to 9.0 and can be used to extract soluble cytoplasmic proteins. However, the preferred pH of the enzyme in this study is pH 6.0; thus, this may be a reason for the low enzyme activity. The components of cell disruption buffers are critical for efficient disruption and will affect subsequent purification steps, including targeting the stability and recovery of the protein. Criteria such as pH, ionic strength, additives to prevent degradation and improve stability, and the buffer-to-cell weight ratio, are required to achieve efficient cell disruption. The pH selected for the lysis buffer should be one pH unit below or above the protein isoelectric point, as this will maintain a positive or negative charge on the protein and prevent isoelectric precipitation^36^. The ionic strength inside the cytoplasm of a typical cell is 0.15–0.2 M, with high concentrations of charged biomolecules available for ionic protein interaction. The lysis buffer should contain at least 0.05–0.1 M NaCl; if the ionic strength of the lysis buffer is increased, it will reduce the ionic interactions^36^. The isoelectric point of XT6 is 9, and therefore at lower pH values, the enzyme will have a positive net charge^22,37^. However, the cell lysis was most effective with 0.05 M sodium phosphate buffer at pH 6.0 compared to Tris-HCl (+/− 8 M urea) at pH 8.0.

The synergistic effect of lysozyme and TritonX-100 has previously been shown to increase the amount of cellular lysis substantially; however, in this study, it was not observed^23^. Lysozymes break down the polysaccharide chains of peptidoglycan, which surround the inner membrane of *E. coli* cells^18^. Gram-positive bacteria can be directly exposed to lysozyme; however, the outer membrane of the Gram-negative bacteria needs to be removed before exposing the peptidoglycan layer to the enzyme. TritonX-100 is a non-ionic detergent that can solubilize the outer and inner membranes of *E. coli* cells^38^. The cost of purchasing lysozymes may be a deterrent to enzymatic lysis, as this additional cost may make the operating costs unfeasible. Thus, sonication of cells resuspended in 0.05 M sodium phosphate buffer (pH 6.0) is recommended for cells lysis in future studies.

### Statistical optimization of recombinant XT6 xylanase production in batch fermentation

#### Screening of significant medium constituents for recombinant XT6 xylanase production

Each row in Table 3 represents one of twelve experiments, and each column has a different variable tested at high (+) and low (−) levels. The data obtained from the PBD runs indicate a wide variation in xylanase activity from 13.97 to 40.06 U/ml across the twelve runs. ANOVA demonstrated that this variation due to the effect of the medium and culture conditions on xylanase production was significant (*p* < 0.05). The *R*^2^, or coefficient of determination, is the proportion of variation in the response attributed to the model rather than to random error^39^. A previous study suggested for a good fit of a model^40^, *R*^2^ should be at least 90%. The determination coefficient (*R*^2^) implies that the sample variation of 94% for xylanase production was attributed to the independent variables, and only about 7% of the total variation could not be explained by the model. The closer *R* (correlation coefficient) value is to 1, the better the correlation between the experimental and predicted values. Here, the *R* value (0.94) shown in Table 4 indicated a close agreement between the experimental results and the theoretical values predicted by the model equation.

**Table 3:** PBD matrix for screening of six medium components.

Variable level							Enzyme activity (U/ml)	
Run no	X1	X2	X3	X4	X5	X6	Observed	Predicted
1	25 (−)	0.4 (−)	3 (−)	1.5 (+)	1 (−)	2.5 (+)	25.88	14.61
2	37 (+)	0.6 (+)	3 (−)	0.5 (−)	1 (−)	2.5 (+)	18.81	11.58
3	37 (+)	0.4 (−)	3 (−)	0.5 (−)	2 (+)	0.5 (−)	28.30	15.41
4	25 (−)	0.6 (+)	3 (−)	1.5 (+)	2 (+)	0.5 (−)	17.82	10.32
5	25 (−)	0.6 (+)	5 (+)	1.5 (+)	1 (−)	0.5 (−)	23.65	12.73
6	37 (+)	0.6 (+)	5 (+)	0.5 (−)	1 (−)	0.5 (−)	20.73	13.56
7	37 (+)	0.4 (−)	5 (+)	1.5 (+)	2 (+)	0.5 (−)	31.36	18.19
8	25 (−)	0.4 (−)	5 (+)	0.5 (−)	2 (+)	2.5 (+)	13.97	8.06
9	25 (−)	0.4 (−)	3 (−)	0.5 (−)	1 (−)	0.5 (−)	40.06	32.70
10	37 (+)	0.6 (+)	3 (−)	1.5 (+)	2 (+)	2.5 (+)	21.35	12.22
11	25 (−)	0.6 (+)	5 (+)	0.5 (−)	2 (+)	2.5 (+)	16.83	8.89
12	37 (+)	0.4 (−)	5 (+)	1.5 (+)	1 (−)	2.5 (+)	27.07	16.47

X1: Induction temperature.

X2: Cell density pre- induction (OD_600 nm_).

X3: Post-induction time.

X4: Yeast extract.

X5: Tryptone.

X6: IPTG.

**Table 4:** Analysis of variance (ANOVA) for six variables by PBD.

	df	Sum of squares	Mean square	F-value	*p*-value
Model	6				
Induction temperature	1	70.71	70.71	3.44	0.1126
Cell density pre- induction (OD_600 nm_)	1	149.46	149.46	7.82	0.0429*
Post Induction time	1	300.63	300.63	14.65	0.0123*
[Yeast extract]	1	19.89	19.89	0.96	0.3701
[Tryptone]	1	26.08	26.08	1.27	0.3108
[IPTG]	1	136.74	136.74	6.66	0.0494*
Residuals	5	102.62	102.62		

df: degree of freedom.

*Significant *p* value at *p* < 0.05.

*R*^2^(mean coefficient of determination) = 0.9419.

**Figure 4:**
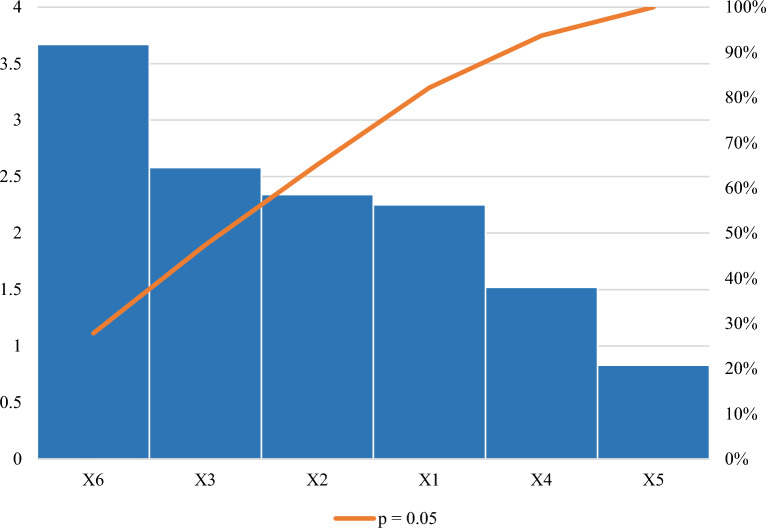
Pareto chart of standardized effects for the production of the recombinant xylanase by *E. coli* BL21. Induction temperature (X1), Cell density pre-induction (OD_600 nm_) (X2), post-induction time (X3), yeast extract concentration (X4), tryptone concentration (X5), IPTG concentration (X6).

The *p* value served as a tool for checking the significance of each of the coefficients, as is shown in Table 4, and indicates that cell density pre- induction (OD_600 nm_) (X_2_), post-induction time (X_3_), and IPTG concentration (X_6_) had a significant effect (*p* < 0.05) on xylanase production. The Pareto chart of standardization illustrated in Fig. 4 confirmed that these three factors significantly influenced xylanase production (*p* < 0.05) as the factors crossed the p-line. However, the other independent factors (*p* > 0.05) were considered insignificant. It has been previously demonstrated in a previous study that the four most relevant variables influencing recombinant protein expression are cell density pre- induction (OD_600 nm_), IPTG concentration, post-induction temperature, and duration of induction^20^.

There was a 94% chance that the model explained the measured variations in response. The magnitude and direction of the factor coefficient in the equation clarified the influence of the six variables for xylanase production. The higher magnitude indicated a large effect on the response. The corresponding response of xylanase production was expressed in terms of the following regression Eq. (6) derived from the Unstandardized Beta values shown in Table 5:

6 $$\begin{aligned} {\text{Y}} & = {\text{ X}}_{{1}} + {\text{ X}}_{{2}} + {\text{ X}}_{{3}} + {\text{ X}}_{{4}} + {\text{ X}}_{{5}} + {\text{ X}}_{{6}} \\ {\text{Y}} & = { 48}.{49 }{-} \, 0.{\text{28X}}_{{1}} - {33}.{\text{37X}}_{{2}} - {3}.{\text{74X}}_{{3}} - {4}.{\text{93X}}_{{4}} - {2}.{\text{35X}}_{{5}} - 0.{\text{98X}}_{{6}} \\ \end{aligned}$$

**Table 5:** Effect estimates for xylanase production from the results of PBD.

Unstandardized beta	Coefficient std. error		Standardized coefficient beta	t-value
Model	48.49	9.57		5.66
Induction temperature (X1)	0.28	0.13	0.36	2.25
OD_600 nm_ (X2)	− 33.37	14.25	− 0.40	− 2.34
Post-induction time (X3)	− 3.74	1.45	0.46	− 2.58
[Yeast extract] (X4)	− 4.93	3.24	− 0.28	− 1.52
[Tryptone] (X5)	− 2.35	2.84	− 0.14	− 0.83
[IPTG] (X6)	− 0.98	0.27	− 0.60	− 3.67

#### Optimization of significant variables using RSM for recombinant XT6 xylanase production

##### BBD

A total of 16 runs were performed to determine the conditions for optimal xylanase production. A matrix was run with the three significant variables as per PBD. The results for the BBD matrix runs in Table 6 showed that run 13 resulted in the highest xylanase activity of 144.02 U/ml under the following conditions: Cell density pre-induction (OD_600 nm_) 0.5, post-induction time of 4 h, and 1.5 mM IPTG, while the lowest activity of 10.18 U/ml was obtained under conditions (cell density pre-induction (OD_600 nm_) of 0.5, post-induction time of 3 h and 0.5 mM IPTG) in run 9. This was markedly higher (*p* > 0.05) compared to the highest enzyme activities obtained during OFAT (16.48 U/ml). Farliahati et al*.*^41^ confirmed a similar influence of optimized parameters on the enhanced xylanase production by recombinant *E. coli* DH5α (1.526–2.655 U/ml).

**Table 6:** Experimental design obtained for the BBD model for three independent variables tested and predicted responses for recombinant xylanase production by *E. coli* BL21.

Run no	Experimental value (Coded value)			Enzyme activity (U/ml)	
	X2	X3	X6	Observed	Predicted
1	0.4 (−)	3 (−)	1.5 (0)	44.72	47.24
2	0.6 (+)	3 (−)	1.5 (0)	10.96	10.45
3	0.4 (−)	5 (+)	1.5 (0)	11.65	10.09
4	0.6 (+)	5 (+)	1.5 (0)	31.42	28.51
5	0.4 (−)	4 (0)	0.5 (−)	13.42	12.55
6	0.6 (+)	4 (0)	0.5 (−)	51.37	50.02
7	0.4 (−)	4 (0)	2.5 (+)	71.59	64.28
8	0.6 (+)	4 (0)	2.5 (+)	26.23	27.68
9	0.5 (0)	3 (−)	0.5 (−)	10.18	9.85
10	0.5 (0)	5 (+)	0.5 (−)	16.24	14.45
11	0.5 (0)	3 (−)	2.5 (+)	101.37	118.01
12	0.5 (0)	5 (+)	2.5 (+)	86.27	86.11
13	0.5 (0)	4 (0)	1.5 (0)	144.02	151.50
14	0.5 (0)	4 (0)	1.5 (0)	127.84	127.47
15	0.5 (0)	4 (0)	1.5 (0)	131.77	126.93
16	0.5 (0)	4 (0)	1.5 (0)	120.72	124.70

X2: Cell density pre- induction (OD_600 nm_).

X3: post-induction time.

X6: IPTG concentration.

For the regression analysis of the experimental data, a quadratic equation was generated for the BBD for optimal xylanase production, as shown in Eq. (7):

7 $${\text{Y}} =\upbeta 0 \, + {\text{X}}_{{2}} + {\text{X}}_{{3}} + {\text{X}}_{{5}} + {\text{X}}_{{{22}}} + {\text{X}}_{{{32}}} + {\text{X}}_{{{52}}} + {\text{X}}_{{2}} {\text{X}}_{{3}} + {\text{ X}}_{{2}} {\text{X}}_{{5}} + {\text{X}}_{{3}} {\text{X}}_{{5}}$$

**Figure 5:**
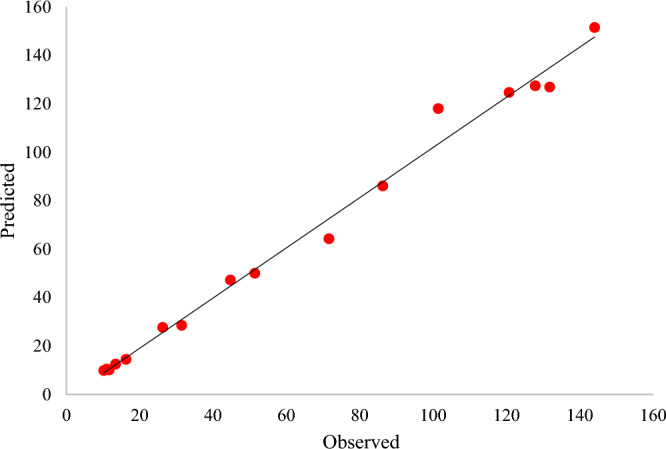
Graphical representation of the minimal difference between the actual (straight line) and predicted responses (circles) for the RSM for optimal recombinant xylanase activity.

The predicted values were determined using the regression equation (Table 6). The *R*^2^ or coefficient of determination (0.9357, close to 1) confirmed the model's validity, i.e., that the model can express 93.57% of the variability of the response. The coefficient of adjusted determination, adjusted *R*^2^, was 0.9383, confirming that the actual values were close to the predicted values^42,43^. The correlation was established by plotting the actual value curve as a function of the predicted values (Fig. 5), which shows the points distributed around the regression line.

Figure 5 shows that the actual response values agreed well with the predicted response values. Thus the predicted xylanase production was within the limits of the experimental factors. Therefore, the model was considered of sufficient quality^42^ with a 93.57% chance that it explained the measured variations in response. Maximum xylanase production (144.02 U/ml) by the recombinant XT6 xylanase occurred in BBD run 13 under optimal conditions (Cell density pre- induction (OD_600 nm_) 0.5, 4 h post-induction time, and 1.5 mM IPTG concentration).

##### Second-order regression and prediction

The second-order regression equation provides the xylanase activity produced by XT6 from *G. stearothermophilus* as a function of cell density pre- induction (OD_600 nm_) (X_2_), post-induction time (X_3_), and IPTG concentration (X_6_) which is presented in Eq. (8):

8 $${\text{Y}} = {5}0.{26 }{-}{ 26}.{\text{75X}}_{{2}} - {2}.{\text{71X}}_{{3}} + {\text{X}}_{{6}} + {\text{ X}}_{{2}}^{{2}} + {\text{X}}_{{3}}^{{2}} + {\text{ X}}_{{6}}^{{2}} + {\text{X}}_{{2}} {\text{X}}_{{3}} + {\text{X}}_{{2}} {\text{X}}_{{6}} + {\text{X}}_{{3}} {\text{X}}_{{6}}$$

where Y is the peak area, X_2_ is the OD_600 nm_, X_3_ is the post-induction time, and X_6_ is the IPTG concentration. The statistically insignificant parameters (*p* > 0.05) and the interactions were omitted from the equation. The model constants and coefficients were generated using the unstandardized beta values.

##### ANOVA and Pareto chart

**Figure 6:**
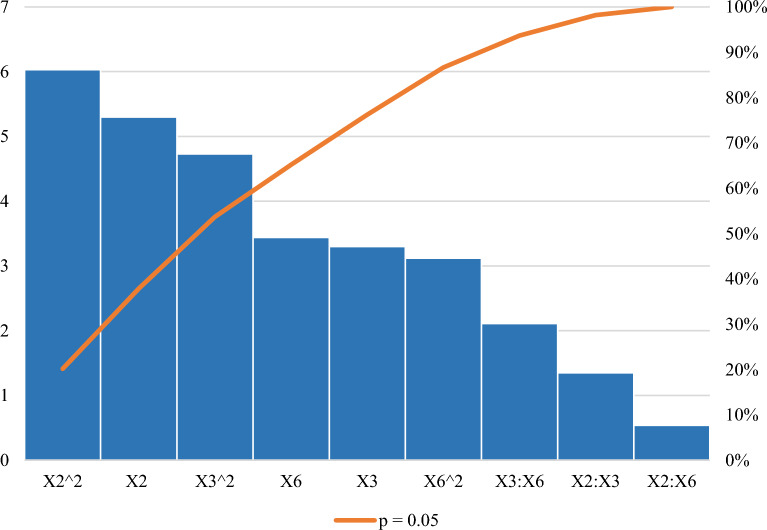
Pareto chart of standardized effects for the nine interactive factors affecting the optimization of xylanase production. Interactions, linear and square terms for cell density (X2), post-induction time (X3), and IPTG concentration (X6).

The “Lack of fit p-value” represented in Table 7, was insignificant as the *p* value was greater than 0.05; in accordance with literature (> 0.05)^44^ and also that significant regression and a non-significant lack of fit of the model were well-fitted to the experiments^44^. The regression equation was validated on this basis^45^ and ANOVA performed to determine the *p* values. As evident in Table 7. This showed the model, the linear and square terms for X_2_ (Cell density pre-induction (OD_600 nm_)), X_3_ (post-induction time), and X_6_ (IPTG concentration) to be significant as *p* values were 0.0018261, 0.0164311, 0.0138398, 0.0009394, 0.0032260 and 0.0207146, respectively. The Pareto chart of standardization histogram graph (Fig. 6) also showed that terms were significant (*p* < 0.05), as the factors crossed the p-line (cumulative % = 50%).

**Table 7:** ANOVA for the response surface methodology parameters for the recombinant xylanase.

Variable	Estimates	Std. Error	t value	*p* value
Model	− 2109.82	364.36	− 5.79	0.0011614*
X2 (OD_600 nm_)	5713.49	1077.59	5.30	0.0018261*
X3 (Post-induction time)	312.47	94.72	3.30	0.0164311*
X6 [IPTG]	241.99	70.27	3.44	0.0137398*
X2:X3	133.83	98.88	1.35	0.2247133
X3:X6	− 208.28	98.88	− 2.11	0.0797899
X2:X6	− 5.29	9.89	− 0.54	0.6119137
X2^2^	− 5963.13	988.86	− 6.03	0.0009394*
X3^2^	− 46.77	9.89	− 4.73	0.0032260*
X6^2^	− 30.80	9.89	− 3.12	0.0207146*

*Significant *p* value at *p* < 0.05.

Adjusted *R*^2^ = 0.9357.

Lack of fit *p* value = 0.0694.

##### Interaction of variables

**Figure 7:**
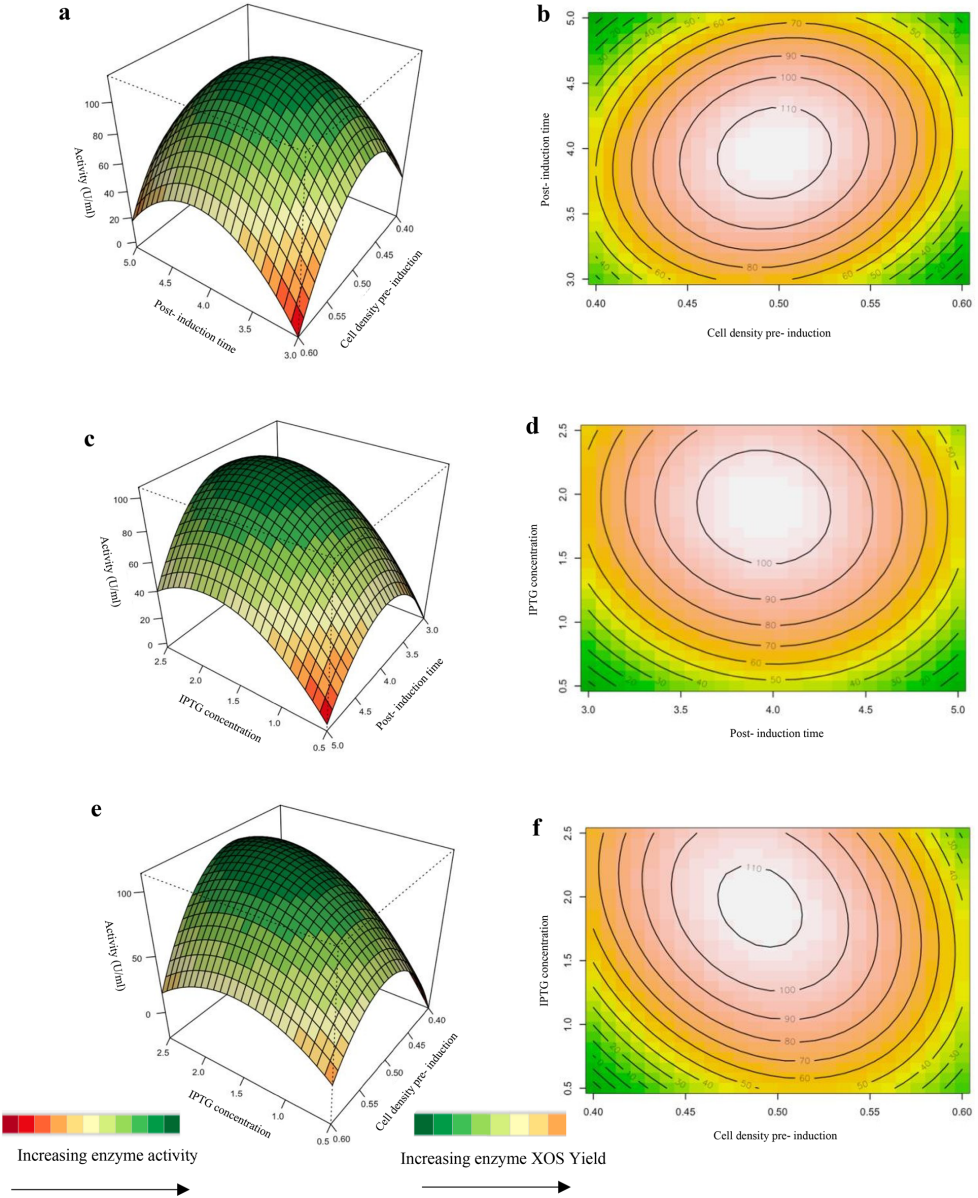
Response surface plots (**a**, **c**, **e**) and contour plots (**b**, **d**, **f**) of the combined effects of; (**a**) and **b**) Cell density pre-induction (OD_600 nm_) (X2) and post-induction time (X3), (**c**) and (**d**): Post-induction time (X3) and IPTG concentration (X6) and (**e**) and (**f**): Cell density pre- induction (OD_600 nm_) (X2) and IPTG concentration (X6) on recombinant xylanase production.

The interaction effects of the variables on xylanase production were also studied by plotting response surface plots and 3D-contour plots against any two independent variables while having another variable at its central level. These plots were drawn to illustrate the combined effect of each independent variable on the response variable and is shown in Fig. 7. The Z-axis refers to the xylanase activity versus any two variables.

###### Effect of cell density pre- induction (OD_600 nm_) and post-induction time

The overexpression of the recombinant XT6 xylanase was influenced by the post-induction time and pre-induction cell density (OD_600 nm_)^20,46^. The interactive effect of cell density pre-induction (OD_600 nm_) (X_2_) and post-induction time (X_3_) was examined, and the results are illustrated in Figs. 7a,b. For this analysis, the other parameter was kept constant at a zero levels. The mutual interaction of both factors (X_2_X_3_) was not significant (*p* > 0.05), indicating that there is no synergistic interaction favoring the expression of recombinant XT6. The highly elliptical response surface plot Fig. 7a shows the highest activity (110 U/ml) of the recombinant XT6 when both variables, cell density pre-induction (OD_600 nm_) (X_2_) and post-induction time (X_3_), were close to the central values, cell density pre- induction (OD_600 nm_) of 0.5 and 4 h, respectively. The findings accentuated that post-induction time was a key factor influencing the expression of XT6 xylanase. In the present study, the post-induction time of 4 h optimally induced production of active recombinant XT6 presumably because this duration was suitable for the correct folding and accumulation of the of recombinant XT6 in *E. coli*. This finding is in accordance with previous reports^20,47,48^.

###### Effect of post-induction time and IPTG concentration

Considering that IPTG is costly and is potentially toxic to cells, it is essential to determine the optimum concentration for induction^49^. The interactive effect of post-induction time (X_3_) and IPTG concentration (X_6_) was examined, and the results are illustrated by both surface and contour plots as illustrated in Fig. 7c,d. The mutual interaction of both factors (X_2_X_3_) was not significant (*p* > 0.05), indicating that there is no synergistic interaction favoring the expression of recombinant XT6 xylanase. The expression of the recombinant XT6 increased with time up to 4 h and IPTG concentration up to its midpoint (1.5 mM), reaching the highest xylanase activity of 100 U/ml.

###### Effect of pre-induction cell density (OD_600 nm_) and IPTG concentration

The dependency of the recombinant XT6 xylanase production on IPTG concentration (mM) and cell density pre-induction is presented in Fig. 7e,f. The interaction between these two parameters was insignificant based on the high *p* value (0.6119) represented in Table 6. At the zero levels (optimal levels) of cell density (OD_600 nm_) and IPTG concentration, the production of recombinant XT6 xylanase will improve as depicted by Fig. 7e. Figure 7f illustrated that with a higher IPTG concentration; the xylanase activity was the highest (110 U/ml) demonstrating that the induction of recombinant XT6 in the middle log phase leads to higher protein expression levels. In this phase, most recombinant bacteria are growing rapidly, and cells are in an ideal environment for the expression of recombinant proteins. A previous study by Batumalaie et al.^20^, also reported that induction at the mid-log phase led to overexpression of lipase KV1 in *E. coli*.

### Scaled-up production of the recombinant XT6 xylanase

Similar or higher enzyme activities are expected when scaling up the production of enzymes from shake flasks to bioreactors^50^. This was observed for the 5-l stirred tank bioreactor run using the same production parameters. The bioreactor maintains a more consistent and homogenous environment with more efficient aeration rates, pH, better mixing, and heat transfer^51^. Table 8 compares xylanase production in shake flask and a stirred tank bioreactor (at different aeration rates). In stirred tank bioreactors, agitation and aeration are essential operational parameters in scaling up aerobic biosynthesis systems and industrial bioprocess development^52^. In aerobic fermentation, the presence of oxygen influences enzyme secretion, which may be attributed to increased metabolic activities of the organism^53^ as has been reported that amylase production by *Bacillus* spp. is strongly affected by the presence of dissolved oxygen.

**Table 8:** Analysis of protein concentration, enzyme activity and specific activity of XT6 produced in batch shake flask and in bioreactor fermentations at different aerations rates.

Samples	Protein concentration activity (mg/ml)	Enzyme activity (U/ml)	Specific activity (U/mg)
Expression in shake flasks	3.01	145.13	48.22
Expression in a 5-l bioreactor (0.5 vvm)	1.57	146.32	93.20
Expression in a 5-l bioreactor (1 vvm)	0.50	165.18	330.36
Expression in a 5-l bioreactor (2 vvm)	1.14	159.44	139.86

Consequently, providing air to the fermentation medium using a compressor under sterile conditions is more efficient by combining agitation with aeration^54^. Higher aeration rates implies improved oxygen supply in the fermenter thus enhanced growth of bacteria and enzyme production. Higher xylanase activities were obtained in the 5-l bioreactor at all the oxygen transfer rates tested [(146.32 U/ml (0.5 vvm), 165.18 U/ml (1 vvm) and 159.44 U/ml (2 vvm)], compared to shake flask studies (145.83 U/ml) under the same production parameters (30 °C, cell density pre- induction (OD_600 nm_) 0.5, 4 h post-induction time, 1% yeast extract, 1.5% tryptone, and 1.5 mM IPTG). Overall, at 1 vvm aeration, the xylanase activity was observed to be the highest (165.18 U/ml). A similar study reported optimal xylanase activity from *Bacillus amyloliquifaciens* at 1 vvm (56.80 U/ml) in a stirred tank bioreactor^48^. Another study showed aeration rates to be a significant factor for high enzyme yields in a stirred tank bioreactor^55^.

#### Effect of aeration rates on pH, dissolved oxygen, biomass, and xylanase activity

**Figure 8:**
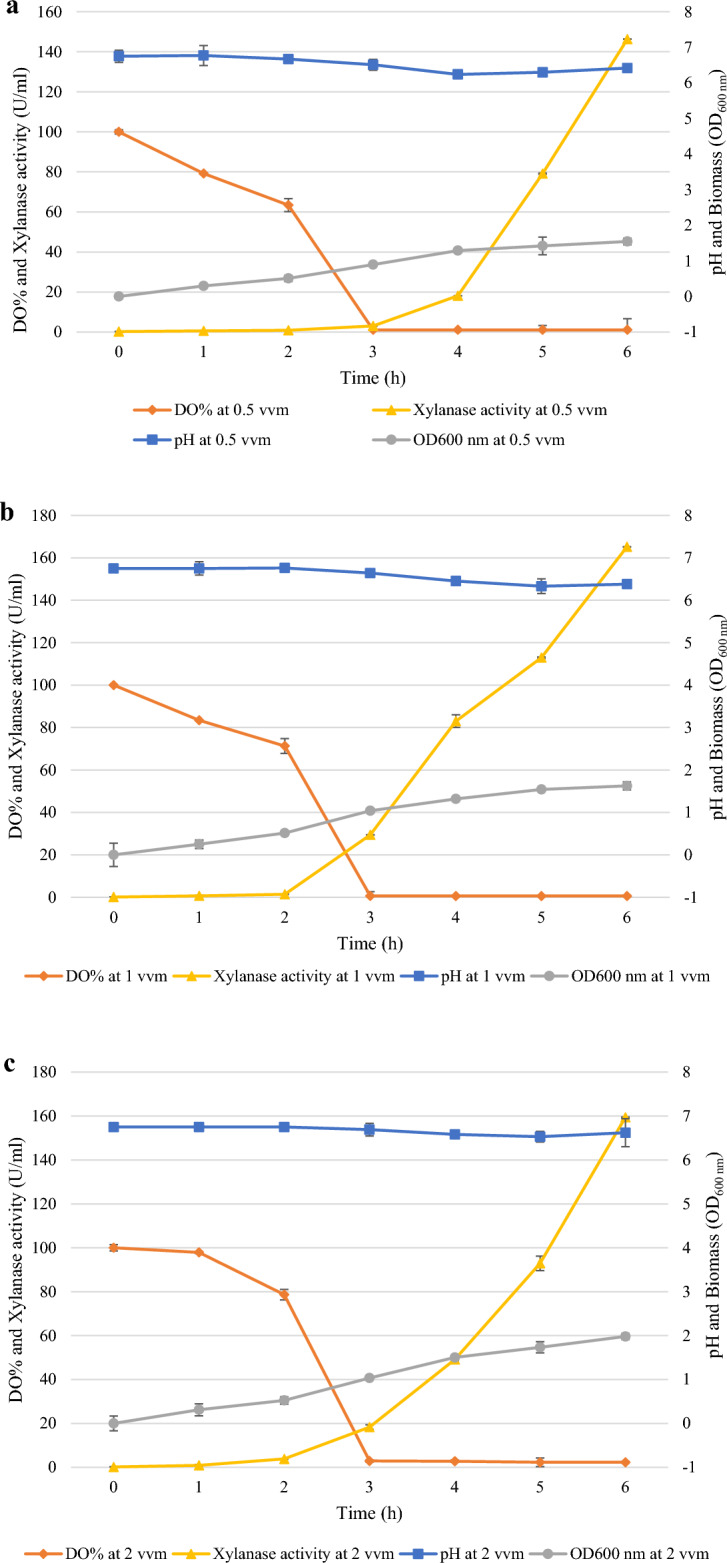
Analysis of pH, dissolved oxygen (DO%), biomass, and xylanase activity at 0.5 vvm (**a**), 1vvm (**b**), and 2 vvm (**c**) aeration rates, during batch fermentation in a stirred tank bioreactor at 200 rpm. 0–2 h represents the time before induction.

Figure 8 shows the fermentation kinetics at 200 rpm and the different aeration rates (0.5, 1, and 2 vvm). Increasing the aeration rate increased biomass, and xylanase production rates and decreased dissolved oxygen. There was more of a change in the fermentation media pH during the growth phase as aeration rates increased. After 6 h, the pH changed from an initial value of 7.00 to 6.42, 6.38 and 6.62 with aeration rates of 0.5, 1, and 2 vvm (Fig. 8a-c), respectively. This can be attributed to higher growth and metabolism rates at the higher aeration rates^56,57^. DO% at 2 h was 63.4, 71.3, and 78.65% at 0.5, 1, and 2 vvm, respectively, and then decreased after 4 h (at 6 h on graph) of expression to 1.1, 0.6 and 2.3% at 0.5, 1, and 2 vvm, respectively shown in Figs. 8a–c. However, as mentioned earlier this had no effect on xylanase activity in the bioreactor. Given the low solubility of oxygen in aqueous solutions^58^, DO in the broth can be limiting, so it is an important influencing factor in aerobic microbial fermentations and can be manipulated up to a point by agitation and aeration rates. The drop in DO levels is due to the active growth phase of the culture when it rapidly consumes oxygen, thus decreasing the oxygen levels in the reactor. While the shake flask culture system allowed oxygen transfer, it was expected to be lower than the 1 vvm achieved in the bioreactor.

#### Effect of aeration rate on specific growth rate, productivity and yield coefficient

Table 9 demonstrates that an increase in the aeration rate resulted in improvements in the specific growth rate, biomass productivity, and yield coefficient. Higher aeration increases the oxygen available to cells, which promotes respiration and the efficient use of the oxidative phosphorylation pathway for energy generation and growth which naturally manifests in a higher growth rate and more significant biomass formation. A change in the aeration rate from 0.5 to 2 vvm resulted in a 1.44-fold increase in the specific growth rate. A similar trend was also observed with the productivity and yield coefficient; however, at 2 vvm, the productivity and yield coefficient decreased. This indicates that the optimum aeration rate for xylanase production in the stirred tank bioreactor is 1 vvm. Similar findings were obtained by Ronda et al.^59^. The highest aeration rate at 2 vvm most likely increased the shear stress on the bacterium, leading to lower biomass productivity and yield coefficient.

**Table 9:** Effect of aeration rates on specific growth rate, biomass yield, and productivity.

Aeration rates (vvm)	Specific growth rate (h^−1^)	Productivity (g/l.h^−1^)	Yield coefficient
0.5	0.25	66	2.71
1	0.27	78	2.82
2	0.36	34	1.28

### Purification of recombinant XT6 xylanase

**Figure 9:**
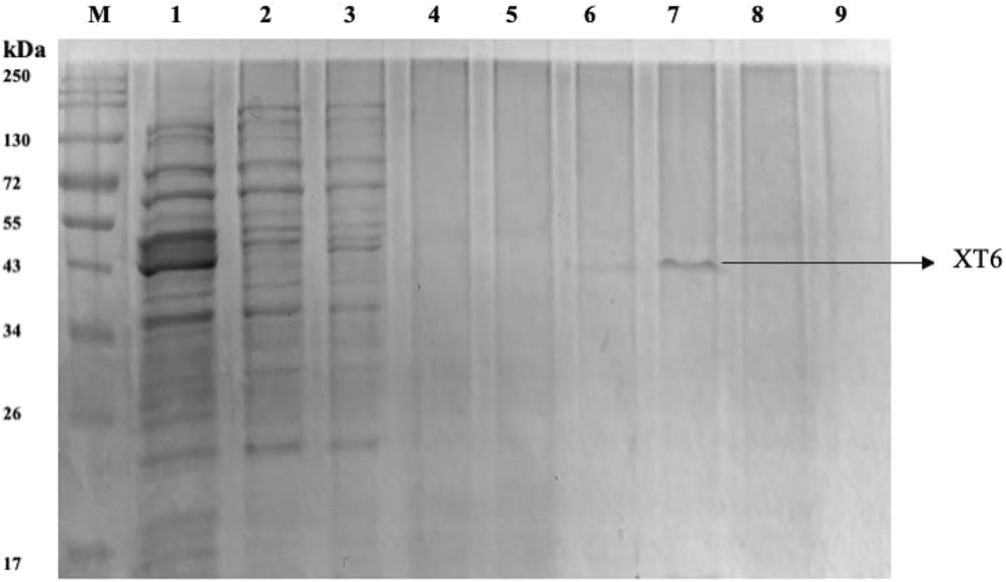
12% SDS PAGE gel of purification fractions of the heterologously produced *G. stearothermophilus* XT6 after affinity chromatography purification in a cobalt column. Lane M: Molecular weight marker (Thermo Scientific, USA), Lane 1: Crude (induced XT6), Lane 2: Flow-through, Lanes 3–5: Wash 1–3, Lanes 6–8: Eluted fractions 1–3, and Lane 9: Wash 4. (Original image shown in supplementary as Fig. 3).

The T-7-based pET vector for the *E. coli* expression system was selected for the production of the recombinant XT6 xylanase, as this expression system has been reported to be fast-growing and produces a high yield of the target protein^20^. This vector is recognized for its expression efficiency and, most importantly, for facilitating purification due to the presence of the His6- tag sequence. The recombinant XT6 xylanase was then purified to homogeneity by HisPur cobalt resin affinity chromatography. SDS-PAGE in Fig. 9 showed that the expressed protein band was 43 kDa in size.

These findings were consistent with a previous study^60^ that optimized recombinant protein expression and purification. The His6-tag sequence aids in the selective binding of the expressed protein to the cobalt beads without impact on protein structure^61^. Therefore, the results from the present study are in accordance with the well-established concept that *E. coli* BL21 (DE3)/pET28a is an excellent expression host/system^15,25,62^. The specific activity of the purified recombinant XT6 xylanase was 4388.55 U/mg (Table 10), 28.97-fold higher than that of the crude lysate (151.50 U/mg) with a 67.04% recovery of the enzyme.

**Table 10:** Purification of the recombinant XT6 xylanase.

Purification step	Total protein (mg)	Total activity (U)	Specific activity (U/mg)	Yield (%)	Fold purity
Recombinant crude lysate	9.50	1440.20	151.50	100.0	1.0
Cobalt column	1.35	1038.15	769.00	72.08	5.08
Ultrafiltration	0.22	965.48	4388.55	67.04	28.97

The higher purification fold (28.97) compared to the recombinant crude lysate shows that the recombinant tagged protein remained in its active conformation^24^. The commercial endo-1,4-β-xylanase (*Bacillus stearothermophilus* T6) has a specific activity of 12–65 U/mg on wheat arabinoxylan, according to Megazyme (CAS number: 9025-57-4). This study yielded a xylanase with a 67.5 to 365-fold higher specific activity (4388.55 U/mg) albeit on beechwood xylan.

## Conclusions

Multiple lysis techniques were tested in this study to release the intracellular protein, including sonication and synergistic lysis with lysozyme and TritonX-100. Based on our analysis, a mechanical technique; sonication of cells resuspended in 0.05 M sodium phosphate (pH 6.0) buffer was recommended for further studies as in resulted in 3.21 fold increase compared to the other lysis techniques. In this study, optimization of the expression of the recombinant XT6 xylanase using PBD and RSM was successfully carried out as these statistical designs showed that under the optimized induction conditions of an induction temperature of 30 °C, cell density pre- induction (OD_600 nm_) at 0.5, post-induction time of 4 h, yeast extract concentration of 1% (w/v), tryptone concentration of 1.5% (w/v), and IPTG concentration of 1.5 mM, the enzyme activity increased from 16.48 U/ml to 144.02 U/ml. The large-scale production of xylanase was successful at 1 vvm aeration and improved the production of the recombinant XT6 xylanase. Compared to commercial XT6 xylanase from megazyme, the specific activity obtained from the scaled-up production is 5.23 fold higher.*.* Future applications involving this enzyme will include testing on animal feed substrates for the production of xylooligosaccharides, which are used as prebiotics in the feed industry, to reduce the feed viscosity and improve the gut microbiota.

### Supplementary Information

Supplementary Figures.

## Data Availability

The datasets used and/or analysed during the current study are available from the corresponding author upon reasonable request. Other data generated or analysed during this study are included in this article [and its supplementary information file].
