# Supplementary material for: Enhanced production of a recombinant xylanase (XT6): optimization of production and purification, and scaled-up batch fermentation in a stirred tank bioreactor
Source: Sci Rep. 2023 Nov 28;13:20895. doi: 10.1038/s41598-023-48202-5 (PMC10684889; doi:10.1038/s41598-023-48202-5)
Supplement: Supplementary file 1 — Supplementary Figures. [file 41598_2023_48202_MOESM1_ESM.docx]

ENHANCED PRODUCTION OF RECOMBINANT XYLANASE (XT6) THROUGH OPTIMIZATION, PURIFICATION, AND SCALE-UP OF PRODUCTION IN A STIRRED TANK BIOREACTOR

**Authors: Priyashini Dhaver^a^, Tariro Sithole^b^, Brett Pletschke^b*^, Bruce Sithole^c,d^, Roshini Govinden^a^**

aDiscipline of Microbiology, School of Life Sciences, Westville Campus, University of KwaZulu-Natal, Durban-4000, South Africa, Email- govindenr@ukzn.ac.za; Tel: 0312608281 bEnzyme Science Programme (ESP), Department of Biochemistry, Microbiology, Rhodes University, Makhanda (Grahamstown), Eastern Cape, South Africa

cBiorefinery Industry Development Facility, Council for Scientific and Industrial Research, Durban 4000, South Africa

dDiscipline of Chemical Engineering, University of KwaZulu-Natal, Durban 4000, South Africa

*b.pletschke@ru.ac.za

M 1 2 3 4 5 6 7 M

Supplementary Figure 1: Original 12% SDS- PAGE gel image representing lanes M: Molecular weight marker (Thermoscientific, USA), l: uninduced fraction, 2-5: induction fractions at 1 to 4 hours, and 6 and 7: BL21 cells without XT6 insert (positive control). Lane M and 1-5 were cropped and represented as Figure 1 in the manuscript.

M 1 2 3 4 5 6 7 8 9


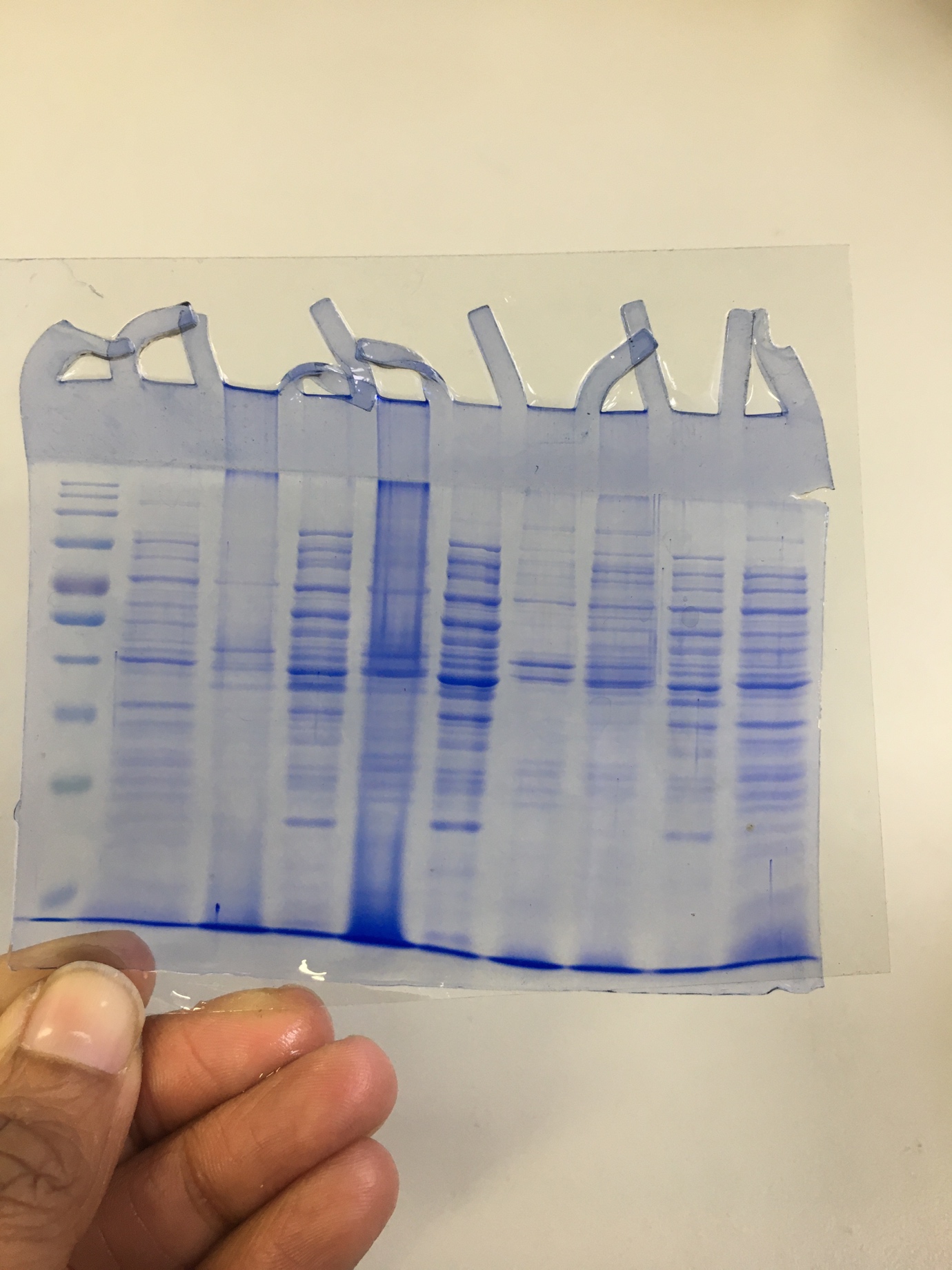


Supplementary Figure 2: Original 12% SDS-PAGE gel image showing expression of the recombinant XT6 in *E. coli* BL21 (DE3) cells after various lysis techniques. Lane M: Molecular weight marker (Thermo Scientific, USA), Lane 1: uninduced sample, Lane 2 and 3: insoluble and soluble fractions tested with lysozyme + 1% TritonX-100, Lane 4 and 5: insoluble and soluble fractions tested with sonication in 0.05 M sodium phosphate (pH 6.0) buffer, Lane 6: insoluble fractions sonicated in 0.05 M Tris-HCl and 8M urea, Lane 7: soluble fraction sonicated in Tris-HCl buffer, Lane 8 and 9: insoluble and soluble fraction with lysozyme. This image was cropped and edited and is represented as figure 2 in the manuscript.

M 1 2 3 4 5 6 7 8 9


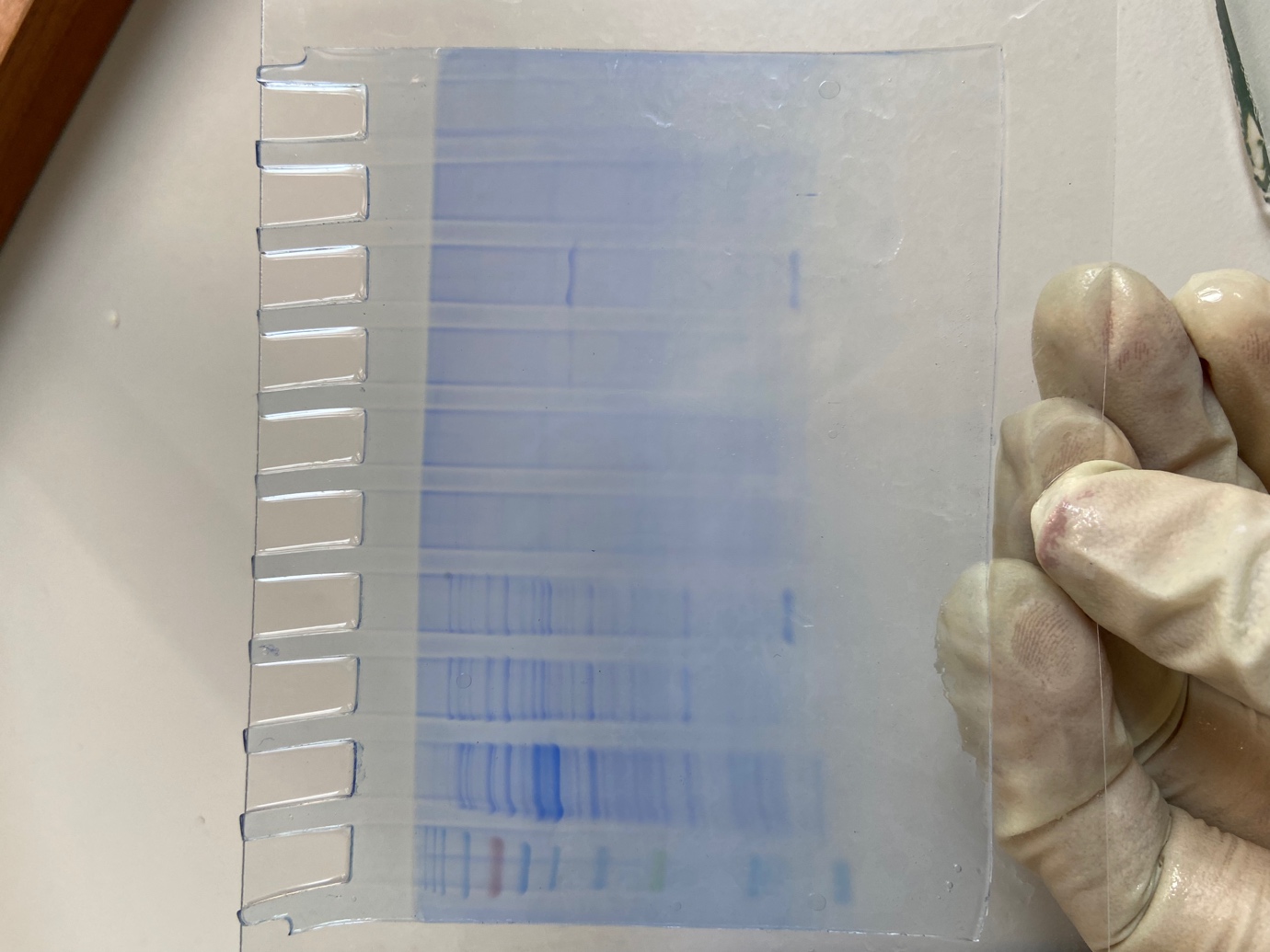


Supplementary Figure 3: Original 12% SDS-PAGE gel image showing purification fraction profiles heterologously produced XT6 after affinity chromatography purification in a cobalt column. Lane M: Molecular weight marker (Thermo Scientific, USA), Lane 1: Crude (induced XT6), Lane 2: Flowthrough, Lane 3-5: Wash 1-3, Lane 6-8: Eluted fractions 1-3, and Lane 9: Wash 4. This image was neatly cropped and edited and shown as figure 9 in the manuscript.
